# Identification of the Most Suitable App to Support the Self-Management of Hypertension: Systematic Selection Approach and Qualitative Study

**DOI:** 10.2196/29207

**Published:** 2021-11-17

**Authors:** Tourkiah Alessa, Mark Hawley, Luc de Witte

**Affiliations:** 1 Biomedical Technology Department College of Applied Medical Sciences King Saud University Riyadh Saudi Arabia; 2 Centre for Assistive Technology and Connected Healthcare School of Health and Related Research University of Sheffield Sheffield United Kingdom

**Keywords:** app, hypertension, self-management, mHealth, blood pressure, support, Saudi Arabia, cardiology, heart, effective, security

## Abstract

**Background:**

Smartphone apps are increasingly being used to aid in hypertension self-management, and a large and ever-growing number of self-management apps have been commercially released. However, very few of these are potentially effective and secure, and researchers have yet to establish the suitability of specific hypertension apps to particular contexts.

**Objective:**

The aim of this study is to identify the most suitable hypertension app in the context of Saudi Arabia and its health system.

**Methods:**

This study used a 2-stage approach to selecting the most suitable app for hypertension self-management. First, a systematic selection approach was followed to identify a shortlist of the most suitable apps according to the criteria of potential effectiveness, theoretical underpinning, and privacy and security. Second, an exploratory qualitative study was conducted to select the most suitable from the shortlist: 12 doctors were interviewed, and 22 patients participated in 4 focus groups. These explored participants’ attitudes towards self-management apps in general, and their views towards the apps identified via the systematic selection process. The qualitative data were analyzed using framework analysis.

**Results:**

In the first stage, only 5 apps were found to be potentially effective while also having a theoretical underpinning and protecting users’ data. In the second stage, both doctors and patients were generally interested in using hypertension apps, but most had no experience with these apps due to a lack of awareness of their availability and suitability. Patients and doctors liked apps that combine intuitive interfaces with a pleasant and clear visual design, in-depth features (eg, color-coded feedback accompanied with textual explanations), activity-specific reminders, and educational content regarding hypertension and potential complications. When the pros and cons of the 5 apps were discussed, 3 apps were identified as being more suitable, with Cora Health rated the highest by the participants.

**Conclusions:**

Only 5 apps were deemed potentially effective and secure. Patients’ and doctors’ discussions of the pros and cons of these 5 apps revealed that 3 out of the 5 are clearly more suitable, with the Cora Health app being judged most suitable overall.

## Introduction

Hypertension is one of the most common chronic diseases in adults, affecting 1 billion people worldwide and causing serious health complications, including stroke, heart disease, and renal failure [1-5]. Among Saudi adults over 30 years of age, 27.2% have been diagnosed with hypertension. Self-management can help control blood pressure (BP), mitigating complications arising from hypertension. However, patients commonly encounter substantial barriers to effectively self-managing their condition [6], and many fail to adequately self-manage their BP [2,4,7].

Smartphone use has expanded in recent years, including in Saudi Arabia, where there were 21.8 million smartphone users in 2018. This has resulted in increased access to health apps, which have the potential to assist patients’ self-management, for example, by providing educational information and self-monitoring tools [8,9].

Alessa et al [10] have shown that smartphone apps with “comprehensive functionalities” are potentially effective. However, relatively few commercial apps meet these criteria [11], and most lack adequate security measures [11]. Inadequate privacy and security lead to potentially unacceptable risks to users’ confidentiality. These authors also found that commercial apps generally lack a clear theoretical basis despite self-management aids having been shown to be more effective when they are theory based [12]. It is imperative that health care only implements interventions that are effective and safe. Privacy, security, and a sound theoretical underpinning should therefore be considered when selecting the most suitable self-management apps.

Although acceptance of an app positively influences its successful use in self-management [13,14], potential users were not consulted about their needs in the development of most of these apps [11,15]. In Saudi Arabia, most hypertension management takes place in hospitals and primary care centers, meaning doctors are the health care workers most actively involved in aiding patients’ self-management [16]. However, very few studies have explored patients’ or doctors’ views toward these apps in general [17-19], and even fewer have examined the Saudi context or that of the other Gulf countries.

The aim of this study is thus to distinguish those hypertension self-management apps that are effective, secure, and underpinned by sound theory, and to identify the most suitable apps for the Saudi context by exploring their acceptance among Saudi doctors and patients. This study will offer a clear approach to selecting effective, secure, and acceptable apps among the many available on commercial app stores.

## Methods

### Study Design

This study consists of 2 stages. The first adopted a systematic approach using criteria of potential effectiveness, privacy and security, and theoretical underpinning to identify potentially effective and secure apps. The second stage consisted of a qualitative study assessing doctor and patient attitudes toward and acceptance of apps that meet these criteria.

### Stage 1: Selection Process According to Existing Evidence

Alessa et al [11] found that 30 hypertension self-management apps out of 186 that were commercially available possess “comprehensive functionalities” and are therefore potentially effective. The present study assessed these 30 potentially effective apps against the criteria of privacy and security and theoretical underpinning.

Privacy and security were assessed based on the Online Trust Alliance [20] and the recommendations of the Information Commissioner’s Office. Apps were assessed by 2 reviewers based on the availability of privacy policies, data collection and sharing practices, and data security [11]. Theoretical underpinning was assessed by the of coding each app according to the behavior change technique (BCT) taxonomy V1 through the identification of the number of BCTs present and their frequency. BCTs were then mapped to the mechanisms of action of the Theoretical Domains Framework [11].

### Stage 2: Qualitative Study

An exploratory qualitative study was conducted to explore participants’ experiences of self-management of hypertension, their attitudes toward self-management apps in general, and their views toward the apps identified via the selection process. This was done via patient focus groups and interviews with doctors. Participants were asked to watch videos providing standardized information about each app and were then asked for their opinions and to rate each app on a 1-to-5 scale (see Multimedia Appendix 1 and 2).

### Participants

The qualitative study was conducted in Riyadh, Saudi Arabia. Convenience sampling was used to recruit doctors and patients [21] at 2 primary care centers and 2 hospitals via posters and flyers. Participants responded by email or phone and were sent an information sheet relevant to their involvement as either a doctor or patient. Suitable times were arranged for the focus groups and interviews. Before the commencement of each of these sessions, participants completed a consent form. The ethical approvals for this study were obtained from the ethical committee of the School of Health and Related Research at the University of Sheffield and the ethical committee of the Saudi Ministry of Health (reference #023341 and #18-56ZE, respectively).

To be eligible, focus group participants had to be 18 years or older, have hypertension as a primary disease for a minimum of 6 months, and be able to speak and give consent. Exclusion criteria were having a cognitive impairment or pregnancy. The eligibility criterion for doctors was having treated patients with hypertension for a minimum of 6 months. Interested participants were sent an information sheet and consent form. The interview and focus groups were conducted by the researcher (TA) in Arabic, which is the native language of participants and the researcher. The transcripts were translated into English by TA and then back translated into Arabic by a professional translation service to ensure accuracy.

### Data Analysis

Descriptive statistics were compiled from relevant quantitative data. All qualitative interviews were recorded, transcribed, and then checked for accuracy against the audio files before being translated. Framework analysis was used to analyze the transcripts using NVivo 12 software (QSR International). Framework analysis consists of 5 stages: (1) familiarization, (2) identifying a theoretical framework, (3) indexing, (4) charting, and (5) mapping [22,23]. Data familiarization was achieved by the researcher (TA) conducting interviews and focus groups, and transcribing and checking the transcriptions.

The analysis framework had 2 parts. The first part concerned participants’ attitudes toward self-management apps in general. The second part of the framework examined participants’ attitudes toward 5 specific apps. The a priori themes and subthemes were confirmed by discussion among the study researchers and summarized. Transcripts were indexed according to these themes and subthemes by TA. If emergent themes and subthemes were identified, TA would add them and recheck the other transcripts for this new theme. The final themes and subthemes were agreed upon through regular discussion between all of the study authors.

## Results

### Stage 1: Selection Process According to Existing Evidence

Table 1 shows the 30 apps previously identified as potentially effective [11]. All were found to have a theoretical underpinning. The BCTs in these apps linked to 10 out of 14 Theoretical Domains Framework mechanisms of action, with the number of mechanisms underlying each app ranging from 5 to 9.

Twenty-two apps were excluded because they did not have an available privacy policy (n=10) or because they insufficiently protected users’ data (n=12).

Of the remaining 8 apps, 3 were duplicates, meaning they were identical versions of the app available for both Android and iPhone platforms. ESH Care (ESH) was also a duplicate, but the Android version had previously been excluded. Only one version of each of the apps was considered, leaving a total of 5 unique apps: Cora Health (Cora), ESH, LifeCourseHyTen (Hyten), Qardio, and Braun Healthy Heart (Braun).

**Table 1:** Privacy, security, and theoretical underpinning of the 30 potentially effective apps.

Number	App name	Version type	TDF^a^ mechanisms of action, n	Privacy and security^b^
1	Blood pressure-Smart BP^c^	iPhone	7	No
2	Fast BP	iPhone	6	No
3	BP Wiz	iPhone	6	No
4	Blood pressure and plus diary	iPhone	7	No
5	BP Grapher simpler	iPhone	7	No
6	BP matters	iPhone	5	No
7	Braun Healthy Heart	iPhone	7	Yes
8	Braun Healthy Heart	Android	7	Yes
9	Qardio	iPhone	5	Yes
10	Qardio	Android	5	Yes
11	Blood Pressure (My Heart)	Android	7	No
12	Blood Pressure Diary	Android	5	No
13	Homedic	iPhone	7	No
14	Hemie	iPhone	4	No
15	LifeCourse HyTen	iPhone	5	Yes
16	LifeCourse HyTen	Android	5	Yes
17	Goal Achiever	Android	7	No
18	Cardio Journal – Blood Pressure diary	Android	6	No
19	Control tension	iPhone	6	No
20	Control tension	Android	6	No
21	ESH Care	iPhone	7	Yes
22	ESH Care	Android	7	No
23	Paracelsus (Pressure control)	Android	7	No
24	Blood Pressure Companion	iPhone	7	No
25	Cora Health	iPhone	9	Yes
26	HeartStar	iPhone	7	No
27	Kang BP	iPhone	6	No
28	BP Diary	Android	7	No
29	BP Diary	iPhone	7	No
30	Bprsseo pro	Android	7	No

^a^TDF: Theoretical Domains Framework.

^b^Apps that meet the criteria for data gathering, sharing, and security have “Yes” indicated, and those that do not have “No” indicated.

^c^BP: blood pressure.

### Stage 2: Qualitative Study

#### Participant Characteristics

Twenty-two patients attended four focus groups, with five to six participants in each group. Twelve doctors were interviewed. The participant characteristics are displayed in Tables 2 and 3.

**Table 2:** Characteristics of the patient's sample (N=22).

Characteristic		Value
**Age (years), mean (range)**		50 (33-74)
	18-30, n (%)	0 (0)
	31-40, n (%)	4 (18)
	41-50, n (%)	6 (28)
	51-60, n (%)	8 (36)
	>61, n (%)	4 (18)
**Gender, n (%)**		
	Males	13 (59)
	Females	9 (41)
**Time since diagnosed with hypertension (years), n (%)**		
	<1	4 (18)
	1-3	6 (27)
	>3	12 (55)
**Education level, n (%)**		
	Less than high school diploma, n (%)	3 (14)
	High school diploma	5 (23)
	Bachelor’s degree	8 (36)
	Master’s degree	4 (18)
	Doctorate	2 (9)
**Smartphone users, n (%)**		
	Yes	20 (90)
	No	2 (10)
**Smartphone brand, n (%)**		
	iPhone	15 (75)
	Android	5 (25)

**Table 3:** Characteristics of interviewed doctors (N=12).

Characteristics		Value
Age (years), mean (range)		40 (28-57)
**Gender, n (%)**		
	Males	4 (33)
	Females	8 (67)
Work experience with hypertension (years), n (%)		15.8 (4-39)
**Profession, n (%)**		
	Resident doctor	2 (17)
	Specialist doctor	6 (50)
	Consultant doctor	4 (33)
**Smartphone owner, n (%)**		
	Yes	12 (100)
	No	0 (0)
**Smartphone brand, n (%)**		
	iPhone	7 (58)
	Android	5 (42)

#### General Views Toward and Experiences of Using Mobile Apps

Table 4 presents the themes and subthemes from the first part of the study framework. This is followed by a description of the results. Selected participants’ quotations are provided in Multimedia Appendix 3.

**Table 4:** Identified themes and subthemes via framework analysis.

Theme and subthemes		Topics
**Self-management experiences^a^**		
	*Strategies used by patients and their compliance*	Adherence to self-monitoring BP^b^, taking required action, adherence to taking medication, adherence to lifestyle, and managing stress
	*Barriers and issues of using strategies for self-management*	Lack of knowledge, busy life, lack of motivation, forgetting, acceptance of disease, asymptomatic patients affecting lack of patient initiative, beliefs about medication, and fear caused by high BP
	Role of doctors	Education about and encouragement of self-management strategies
	*Patient knowledge and awareness about hypertension*	Current patient knowledge and required information
**Using health apps for self-management**		
	*Doctors and patients experience in using health apps*	Patients’ experiences in using general apps and HTN^c^ apps, and doctors’ experiences in using health apps or recommending HTN apps
	Expected useful features of smartphone apps	Self-monitoring and reminders, educational information, and feedback
	*Factors affecting uptake of the app*	Demographic factors including age, education, and IT^d^ literacy; app usability, app’s language, and doctor support
	Concerns about using health apps for self-management	Credibility and accuracy, company intentions, patient commitment in using the app, and app usability

^a^Italics indicate a priori themes.

^b^BP: blood pressure.

^c^HTN: hypertension.

^d^IT: internet technology.

#### Self-management Experience

The majority of doctors noted that most patients take their medication frequently, but some fail to monitor and record their BP. Most patients reported that they tried to monitor their BP and take medication regularly, and tried to stay healthy through diet, exercise, and managing stress. Patients and doctors acknowledged the role of doctors in encouraging patients to effectively self-manage their condition, for example, by setting strategies and goals together, and encouraging patients’ adherence to these.

Several barriers to patients’ involvement in self-management were mentioned. Doctors identified lack of patient initiative, acceptance of the disease, and inaccurate negative beliefs about medication as the most common barriers. However, patients reported barriers such as relying on impractical tools to record data, lack of knowledge relating to hypertension management, lack of motivation, forgetting, busy lifestyle, social pressures, and lack of exercise opportunities.

Doctors and patients believed that lack of patient knowledge negatively affected self-management. Doctors also expressed concern about patients accessing inappropriate or incorrect information. Doctors felt that younger patients and more educated patients tended to be better informed but would not necessarily take greater responsibility for their own health due to a lack of determination or concern.

#### Using Health Apps for Self-management

Most doctors reported having experience of using health apps themselves. Patients had experience of using apps for nonmedical purposes (eg, entertainment, socializing) but only 1 patient had ever used a hypertension self-management app before. The other patients were unaware of their availability or suitability. Doctors also had never recommended health apps to their patients. However, the data showed that participants were generally interested in using hypertension apps to support self-management and expected that these would have useful features, such as self-monitoring of BP.

Among doctors, users’ ages and educational levels were considered the most influential factor affecting use of hypertension apps, whereas for patients, the most important factors were app language and usability.

Doctors expressed concerns about the credibility and accuracy of the apps, and doubt about their continued availability. They felt that they would be more willing to recommend apps that had been scientifically tested, were based on practice guidelines, or had been checked by doctors.

#### App Preference

Table 5 presents the themes and subthemes from the second part of the study framework. This is followed by a description of the results (a table showing the side-by-side data for each of the 5 apps is presented in Multimedia Appendix 4).

**Table 5:** A final framework developed to evaluate 5 apps after completing the analysis process.

Theme and subthemes			Topics
**Adequacy of app content^a^**			
	*User data collected*	Accuracy and method of data inputting, and type of data collected	
	*Feedback and tracking progress*	Presentation of feedback and accuracy of feedback	
	*Reminder*	N/A^b^	
	*Information provided*	Level of details and type of information (information topics)	
	Social Support	Communication with others	
	Content credibility	Credibility	
**App usability**			
	*How easy to use*	App design, layout, and navigation	
	*Training*	Type and intensity of training required	
**Overall app assessment**			
	Factors affecting uptake and usage	Demographic factors including, age, education, and IT^c^ literacy; app feature; language; price; privacy; and ads and promotion	
	Rating and recommendation	App rating, doctors’ willingness to recommend apps, doctors’ estimated uptake, patients’ willingness to use and recommend apps, general recommendations	
**Potential benefits and drawbacks of app use**			
	Expected risks of inappropriate content	Difficulties, including stress, anxiety, and confusion; and decreased app use and poor self-management	
	Support patients’ self-management	Controlled BP^d^, empowered self-management, improved compliance and knowledge, and supportive doctors	

^a^Italics indicate a priori themes.

^b^N/A: not applicable.

^c^IT: internet technology.

^d^BP: blood pressure.

#### Adequacy of App Content

##### Feedback and Tracking Progress

Most doctors and patients liked the 5 apps’ method of presenting data in different formats, such as in graphs and tables. The data showed a preference for apps with high-quality graphs (Cora and ESH care), for feedback that used color coding and supplementary text (Cora), and for the automatic calculation of BMI (Qardio and ESH) or BP average (Qardio, ESH, and Cora). Doctors thought that the feedback of all of the apps could be improved if it offered the feature of setting goals (eg, for BP) that was tailored to patients’ circumstances and demographic.

##### Reminders

Participants liked the reminder feature for self-management activities in all 5 apps. Both doctors and patients preferred apps, like Cora, that provide reminders for different tasks (eg, self-monitoring of BP) over apps that provide only a reminder for medication (ESH and Hyten) or a generic reminder for a nonspecified task (Braun and Qardio). A few doctors liked apps that allowed reminders for different medications and doses (ESH and Hyten).

##### Information Provided

Participants found Qardio’s lack of educational information unhelpful. Opinions varied as to the usefulness of information offered by the other apps. Doctors generally criticized apps, like Braun and ESH, that lacked any information about medication and side effects, but also felt that detailed information about side effects of medication (Hyten) might be off-putting for patients. Participants thought that apps (eg, Cora) that have information about hypertension in general, as well as data on hypertension risks, BP readings, and how to measure BP, would benefit patients.

##### User Data Collected

Participants favored apps that collected detailed information that had easy and clear methods of data entry. They preferred apps that collect other data in addition to BP, such as exercise (Cora and Braun). They felt that some apps are not detailed enough to capture all relevant information (eg, entering the type of exercise) and found the way of entering data in some apps to be more difficult than that in others (Braun), not well organized (Hyten), or likely to lead to typo mistakes (ESH).

##### Social Support and Content Credibility

Patients had mixed opinions about the social support feature. Some found it useful while others found it unhelpful or unnecessary, given the increased access to social media platforms. Doctors felt that the credibility of educational information should be ensured, either by assessing if the information was based on medical guidelines or by having apps reviewed by other doctors or medical companies. One doctor suggested that profit-motivated app development may not lead to the best quality information being included.

#### App Usability

Participants preferred interface designs with easy and clear layouts, where features of the app are easy to reach (eg, with app functions visible in the main menu like in Cora and ESH) rather than embedded in other functions (Qardio and Braun Health).

The muted color schemes of Hyten, Cora, and ESH were considered more user-friendly than were those with strong, bright colors (Braun).

Most doctors and patients thought that some level of training would be required for all 5 apps although they disagreed over the length and intensity that would be needed.

#### Potential Benefits and Drawbacks of App Use

Participants expressed several possible benefits of using these apps. They thought that reminders and monitoring would help to increase their engagement and that educational information could help to increase their awareness of their condition. However, some doctors were concerned that apps with too few functions (eg, Qardio and ESH) may lead to patients becoming bored, or, conversely, that too much detail (Hyten) or a poor layout (Hyten and Braun) would confuse patients.

#### Overall App Assessment

##### App Rating and Recommendation

The doctors’ and patients' full rankings for all of the 5 apps, which was calculated by aggregating each group’s 1-5 ratings. Cora was ranked highest by both doctors (total 51, mean 4.25) and patients (total 97.5, mean 4.4). Hyten was second among doctors (total 43, mean 3.5), while ESH was second among patients (total 85.5, mean 3.6). ESH was third among doctors (total 41.5, mean 3.4), while Hyten was third among patients (total 80, mean 3.8). Qardio and Braun were ranked lowest by patients (total 64, mean 2.9) and doctors (total 30, mean 2.5), respectively.

Doctors and patients made some recommendations for improvements of app features and content. Cora received the fewest suggestions. Some of the recommendations were common for all 5 apps, such as for the tracking of hospital appointments and other medical conditions. The suggestions are presented in full in Multimedia Appendix 4.

##### Factors Affecting Uptake and Usage

Doctors and patients identified different factors that may affect the use of the 5 apps. Age was a factor mentioned by several doctors who felt that 2 apps (Cora and Hyten) in particular may pose difficulties to older users. Some doctors stressed the importance of official endorsement by, for instance, the Ministry of Health, or public health campaigns to encourage patient uptake. Inexperience with smartphone technology was seen as another major potential barrier. Participants also mentioned the unavailability of apps in users’ own language. Most patients did not express concern with privacy of the apps, but this was mentioned by doctors, particularly concerning high-profile individuals. Patients also expressed concern over app prices and the payment methods that might be required.

## Discussion

### Principal Findings

This study aimed to identify the most suitable hypertension app in the context of Saudi Arabia and its health system using a 2-stage approach: a systematic selection approach that assessed apps according to the criteria of potential effectiveness, theoretical underpinning, and privacy and security; and an exploratory qualitative study involving 12 doctors and 22 patients. The first stage found that only very few apps were deemed potentially effective and secure. The second stage showed that doctors and patients were generally interested in using hypertension apps. Their discussions of these 5 apps’ pros and cons revealed that 3 out of the 5 are clearly more suitable, with Cora being judged the most suitable overall.

### Comparison of the Study Findings With the Literature

The selection approach found that of the 30 apps previously identified as potentially effective [11], all 30 contained a theoretical underpinning but only 5 contained adequate privacy and security measures. This demonstrates the pitfalls of commercial app availability: most apps are unlikely to be effective and secure, leading to potentially serious effects on users’ health and well-being. This suggests a lack of collaboration between researchers, experts, and developers, which would otherwise help in improving the potential effectiveness and quality of apps or provide clear evidence of effectiveness and safety [23-25].

The qualitative study found that both doctors and patients were interested in using hypertension apps but that most had never used these apps or been recommended them, due to a lack of awareness of their availability and suitability. This is in line with previous research, including that of Morrissey et al [18] who found that few hypertension apps were used by patients due to a lack of knowledge of these apps. This highlights the importance of identifying the most suitable apps and raising awareness of these among health care professionals and the public through official media and education channels [26].

Morrissey et al [18] and Vo et al [13] found that some patients expressed no interest in developing the digital competence required to use mobile health (mHealth) interventions. This contrasts with our study, which found that the majority of participants were keen to engage with self-management apps. One possible explanation for this is the relative age of the study populations. In Saudi Arabia, the average age of hypertension sufferers is lower than that in Europe, meaning the study population recruited for our study also had a younger average age and so was likely to have higher digital competence and greater willingness to engage with smartphone technology. Moreover, most participants in this paper had a higher education level and therefore were likely to have high digital competence. Bol et al [27] found that those with a higher level of education were more likely to engage with mHealth interventions than were those with a lower education level. A number of patient participants for this present study had some preexisting medical knowledge, which may also partly explain the relatively high level of engagement.

When the pros and cons of the 5 apps were assessed, 3 apps were identified as being more suitable, with Cora rated the highest in participants’ ratings. Patients and doctors liked these apps because they combine intuitive interfaces with pleasant and clear visual design, in-depth features (eg, color-coded feedback accompanied with textual explanations), activity-specific reminders, and educational content regarding hypertension and potential complications. Apps are more likely to be used and accepted if they include key components, such as pleasing visuals and the facility to personalize, and if they offer other broader functions, such as education [28]. Detailed features allow users to tailor the app to their circumstances and needs, and provide depth of information to support them [13]. Studies have found that apps that are designed to be easy to use lower the effort a user has to expend in using them [13,26], which could explain why users did not prefer the more complex apps. Our study’s findings are also in line with those of Leong et al [29], who found that hypertension apps with an educational component scored higher on the study’s quality checklist compared with those that did not.

Doctors and patients expressed somewhat different concerns in identifying the most suitable apps, with doctors generally being more concerned with medical accuracy and patients being generally more concerned with usability, interface, and visual design elements. This is similar to the findings of previous research revealing that doctors and patients often showed somewhat different priorities or preferences regarding mHealth apps even if they agreed to some extent on which is the best overall [30].

The self-management strategies identified in this present study were largely in line with those identified by Barlow et al [31]. Patients try to adopt a variety of self-management methods to stay healthy, such as self-monitoring BP and doing exercise. However, they face difficulties and barriers that affect or delay the adoption of these strategies. Lack of motivation, a busy life, lack of knowledge, and forgetting, were found to be the most common barriers to self-managing hypertension, which again is in line with other studies, including those specific to the Saudi context [6,30,32]. Although stress and anxiety have been identified as 2 of the most common barriers to effective self-management [6], these were not identified as significant barriers in this study. This may be because the main focus of approaches to self-management is on behavioral and medical management, with less focus placed on assisting patients in dealing with the emotional effects of chronic disease [33]. Participants may therefore not have been primed to discuss these topics. A meta-review found supporting self-management interventions with different components, including self-monitoring BP and provision of information, could be effective in controlling BP and improving adherence to adopted strategies [34]. Khatib et al [6] indicated that the barriers patients identified show that they have an interest in finding a solution to effectively self-manage their hypertension, and these authors call for a more targeted, multifaceted intervention to mitigate the identified barriers affecting self-management. Our study found that patients do indeed have an active interest in using mHealth interventions to support their self-management of hypertension, provided certain barriers can be overcome.

Previous research has shown that despite the many advantages of using apps in supporting self-management, certain concerns regarding their use persist, such as the accessibility and usability of the app and the effectiveness of these tools [13,18,19]. Our data are in line with these previous findings. Some participants felt that apps could be a helpful tool and felt motivated by functions that allowed them to track the entered data and their progress over a long period of time. Both patients and doctors raised concerns about the apps, including about the language, with patients also raising concerns about the apps’ usability. App developers should consider the cultural preferences of target users (eg, language) and their technical preferences (eg, ease of use) to ensure the acceptance of and engagement with their apps in the future and to alleviate any hindrance affecting the use of health apps [13].

Previous research has found that doctors are in general less positive than are patients regarding the use of mHealth apps [35]. In this study, doctors were generally positive about the prospect of their use. However, they were generally more concerned than were patients about the credibility of the app and patients’ ability to continue using it. They also questioned whether older users, who they felt are less competent users of the technology, can easily engage with these apps. Indeed, users’ continued or ongoing use of apps and the credibility of health apps have become a major concern in recent years [13]. Vo et al [13] have suggested that app credibility could be increased if certain standards were developed to ensure that they only provide accurate and evidence-based information. Age and digital competence will become less of an issue as younger users, who have been immersed in smartphone culture, carry this competence with them into their old age. Meanwhile, the provision of training for new or older users could further mitigate these concerns [36,37].

### Strengths and Limitations

The main strength of this paper lies in its development of a rigorous selection approach to identify the most suitable hypertension app(s), which has the potential to be transferred to apps targeting other conditions and in different contexts. There may be some limitations regarding the generalizability of these results. The study used a self-selecting sample of patients. Those who are more interested in and therefore probably more competent with smartphone technology might have been more likely to volunteer, and this might have impacted the results. A number of the patient participants had some preexisting medical knowledge, which may make the findings less generalizable. The number of older participants in the study sample was relatively low, which may further impact the generalizability, especially since the majority of those with hypertension are older people. The selection approach focused on privacy, security, and theoretical underpinning because these criteria were considered as the most important in implementing and using interventions in the health care field. We did not consider other issues such as engagement due to the lack of available information about them. Finally, because none of the identified apps were available in Arabic, standardized video presentations were used to demonstrate how the apps worked, but this might have created a biased presentation of the apps’ functionalities.

### Conclusions

This study found that only 5 apps out of 30 could be deemed potentially effective and secure. It was also found that participants were favorable toward the idea of using health apps to aid in the self-management of hypertension. Through patients’ and doctors’ discussions of their pros and cons, 3 apps were identified as more suitable than the others, with the Cora Health app being the most suitable overall. In a next step, this app should be evaluated for its usability and effectiveness.

## Appendix

Multimedia Appendix 1 Interview topic guide.

Multimedia Appendix 2 Focus group topic guide.

Multimedia Appendix 3 Qualitative data.

Multimedia Appendix 4 App preference.

## Abbreviations

BCT: behavior change technique
BP: blood pressure
Braun: Braun Healthy Heart
Cora: Cora Health
ESH: ESH Care
Hyten: LifeCourseHyTen
mHealth: mobile health
