# Supplementary material for: Identification of the Most Suitable App to Support the Self-Management of Hypertension: Systematic Selection Approach and Qualitative Study
Source: JMIR Mhealth Uhealth. 2021 Nov 17;9(11):e29207. doi: 10.2196/29207 (PMC8663499; doi:10.2196/29207)
Supplement: Multimedia Appendix 1 [file mhealth_v9i11e29207_app1.pdf]

<u>Interview guide for doctors</u>

Personal details:

1. Age
2. Gender
3. Profession
4. Working experience in years
5. Smartphone owner? (Yes, No).
6. What is your smartphone brand?

Discussion Questions:

Black-question to be ask participants, Blue- possible probes and my idea that may explored further.

1. In your experience, how do your patients manage/self-manage their hypertension?

    a. *How do patients monitor their blood Pressure? (if applicable), provide details about the approach they use*
    b. *Do you encourage them to adopt other self-management strategies or skills to control their blood pressure (e.g., healthy diet… etc). Why?/Why not?*

2. In your experience do you know whether your patients have problems with any of the following: Self- monitoring your blood pressure, lake awareness/understanding of managing hypertension and its risk factors, taking medication, diet, exercise, remembering appointments, communication with your doctors? Provide details.

3. Could you please tell me about your **experience** of using smartphone applications (”apps”)?

    a. *Have you ever recommended an app for your patients, for any purpose? For hypertension? Provide details.*
    b. *What were the reasons for recommending the hypertension app that you mentioned? If applicable*

4. What do you feel are the potential benefits for patients of using smartphone apps to support the self-management of their hypertension?

5. Do you think there are any limitations in or barriers to using smartphone apps to support the self-management of hypertension?

a. *Are there any other limitations that need to be considered?*
b. *Do you have any concerns you had about the use of smartphone apps in the self-management of hypertension?) please explain.*

The doctor will be given a demonstration of the five apps supporting the self-management of hypertension. The video will then demonstrate how each app works and its functionalities, such as self-monitoring blood pressure, reminders and feedback. The following questions will be asked for each app after stopping the video:

6. Could you describe in as much detail as possible how satisfied you would be for your patients to use the apps? Why? (Please provide reasons for your answer).

    a. *Do you think it would be useful for patients? And why?*
    b. *How easy or difficult it would be for patients to self-manage hypertension by using the app?*
    c. *How confident do you feel that your patients could successfully self-manage their hypertension by using this app*

7. What are the potential benefits you might expect for your patients using this app in support the self-management of hypertension?

8. Do you think there are potential limitations in, or barriers to, using this app to support the self-management of hypertension?
    a. *Do you have any concern about the use of smartphone apps in your home? And why?*
    b. *How important is it for you to check the credibility of a smartphone app's information source (content source, who created it)?*

9. How likely do you think your patients would you be to use a smartphone app? Please explain your answer.

    a. *Are there any other features that you think should be considered to increase patients' willingness to use a smartphone app? and how patients can effectively benefit from it?*

10. To what extend you are willing to recommend this app for your patients?

11. How likely patients may need training to use such a smartphone app? If so, please provide details.
12. Do you have any other questions about the app?

13. Please rate this app from 5 (very suitable) to 1 (not suitable) for self-managing hypertension.
