# Supplementary material for: Identification of the Most Suitable App to Support the Self-Management of Hypertension: Systematic Selection Approach and Qualitative Study
Source: JMIR Mhealth Uhealth. 2021 Nov 17;9(11):e29207. doi: 10.2196/29207 (PMC8663499; doi:10.2196/29207)
Supplement: Multimedia Appendix 2 [file mhealth_v9i11e29207_app2.pdf]

Black-questions to be ask participants, Blue- possible probes and my idea that may explored further.

1. In your experience, how do you manage/self-mange your hypertension?

    a. *How often do you check your BP?*
    b. *What actions if any do you take in response to your blood pressure Readings?*
    c. *What other self-management strategies or skills have you adopted to control your blood pressure, and how? (for example, trying to eat a healthy diet, or taking exercise).*
    d. *What problems are you facing in adopting self-management strategies? Please provide details.*
2. How knowledgeable are you about hypertension, e.g. what the risks are?

3. Can you please tell me about your **experience** of using mobile applications (e.g.,"apps")?
    a. Have you ever used an app for any purpose? Provide details.
    b. What were the reasons for using the app that you mentioned? If applicable

4. Have you ever used an app for managing your hypertension? Why/Why not?

5. What do you feel are the potential benefits of using mobile apps to support the self-management of hypertension?

6. What are the potential limitations of, or barriers to, using mobile apps to support the self-management of hypertension?

    a. *Can you tell me about any concerns you had about the use of mobile apps?*

The patients will be given a demonstration of the five apps supporting the self-management of hypertension. The video will then demonstrate how each app works and its functionalities, such as self-monitoring BP, reminders and feedback. The following questions will be asked for each app after stopping the video:

7. Could you describe in as much detail as possible how satisfied you are with the app? Why?
    a. *How useful did you find it? Why/Why not?*
    b. *How easy was it to use the app to self-manage hypertension?*
    c. *How confident do you feel that you could successfully self-manage your hypertension using this app?*

8. What are the potential benefits you might expect from using this app to support the self-management of hypertension?

9. What are the potential limitations of, or barriers to, using this app to support the self-management of hypertension?
    a. Can you tell me about *the use of mobile apps in your home? Please explain*
    b. *How important is it for you to check the credibility of a mobile app's information source (content source, who created it)? If applicable*
10. How likely would you be to use a mobile app? Please explain your answer.
    a. *Are there any other features that you think should be considered to increase your willingness to use a mobile app? What they are? and how would they help you?*

11. Would you recommend this app for your friends or families? Why/Why not?
12. Do you think you may need training to use a mobile app like this? Why/why not?
13. Do you have any questions about the app?
14. Please rate this app from 5 (very suitable) to 1 (not suitable) for self-managing your condition.
