# Supplementary material for: Identification of the Most Suitable App to Support the Self-Management of Hypertension: Systematic Selection Approach and Qualitative Study
Source: JMIR Mhealth Uhealth. 2021 Nov 17;9(11):e29207. doi: 10.2196/29207 (PMC8663499; doi:10.2196/29207)
Supplement: Multimedia Appendix 3 [file mhealth_v9i11e29207_app3.pdf]

Theme/Subtheme	Quotation
**Self-management experience**	
Strategies used by patients and their compliance	*"[…] only 50% - 60% that we ask to take their BP regularly actually do so" [D2-male]*<br><br>*"[…] with some older people, we found that their children had recorded their BP reading in a table format. They were very committed to helping their parents" [D1-male]*<br><br>*"I try to manage my hypertension as much as I can. I self-measure my blood pressure when I feel symptoms like dizziness." [Focus group2-male]*<br><br>*"I tried to self-monitor my blood pressure regularly and take medications, but I am busy with my daily work that negatively affect my adherence." [Focus group3-male]*<br><br>*"The doctor always asked me about monitoring my blood pressure at every visit, but I totally forget to do that because my blood pressure is a little bit controlled" [Focus group2-male]*<br><br>*"When it [BP] is high I tried to do anything to reduce it. For example, I sometimes go walking or even take an extra pill to reduce it" [Focus group1-male]*<br>*"For me, when it [BP] was extremely high I call 973 which I can speak to a doctor to help me and give me advices" [Focus group1-female]*<br><br>*"I try to walk every day and make it part of my routine, but mostly I do not walk much and only remember later that I should have... I need something to help encourage me to do it frequently" [Focus group 4-female]*

Patient knowledge and awareness about hypertension	*"People lack knowledge about hypertension; patients don't know the type of food that they eat and if they are good or not, what causes blood pressure, hypertension complications, and so on."[D11-female]*<br><br>*"We need more information on how to measure blood pressure correctly, hypertension risks, and the medicines, but the information in Arabic is not always reliable." [Focus group 2-female]*<br><br>*"I have a problem when I measure as I do not know if it is high or normal, even though the doctors always told me. Is there any information to help me understand the normal ranges?" [Focus group2-male]*<br><br>*"In Saudi Arabia, many people know about hypertension, and the benefits of making positive lifestyle changes like exercising. Some people don't take it seriously, as the effects of the diseases aren't necessarily present at the time of the diagnosis, but will appear later and cause complications, such as high blood pressure and diabetes. When a patient has a clot or something else, some think this is not related to hypertension, even though we told them about this information before" [D1-male]*<br><br>*"It sometimes causes problems when people worry about the wrong information that they have found on the internet. I advise people that not everything that they read on the internet and discussion groups is correct, and to only consult their doctor or a medical website" [D10-female]*

Role of Doctor	*"When I was diagnosed with hypertension, I agreed with my doctors on the goals, such as walking, running, and the amount of appropriate food." [Focus group1-male]*<br>*"We give them different activities to do such as exercises, usually they are old-aged people, so we advise them to walk for half an hour a day. I then start to increase their awareness about what affects BP and their general health education so they know how important it is. We encourage them and some do their best." [D10-female]*
Barriers and issues of using strategies to self-management	*"Patients usually did not take their hypertension seriously" [D6-female]*<br><br>*"They do not know they are chronic diseases and should have taken medication for a long time." [D8-female]*<br><br>*"I sometimes self-measure my blood pressure, but sometimes I forget or because of busy daily life." [Focus Group1-female]*<br>*"In my opinion, I feel hypertension is a simple disease, it does not need a serious care. If my blood pressure raise what is the problem" [Focus Group1-male]*<br><br>*"Nothing helps me, even putting paper next to my bed to remember before going to sleep, because I will be tired and forget to do anything. "' [Focus Group2-female]*<br><br>*"Using paper is not practical because a paper or pen is not always available, and it is not very reliable either." [Focus Group2-male]*
**Using Health apps for self-management**	

Doctors' and patients' experiences of using smartphone apps	*"I had no idea that there were apps that I could recommend to patients - I only use health apps that help me as a doctor to calculate scores, and choose satisfactory ways to prescribe the appropriate dose, as well as decision support apps, but I have not used them at the level of patients." [D3-female]*<br><br>*"I haven't recommended apps specifically for patients - generally, we don't know if the app is useful. This is the main aspect: we don't know if monitoring apps helps patients or not, unless there is a study proving that they are useful." [D2-male]*<br><br>*"Yes, I used an app for blood pressure, I forget its name." [Focus group2-male]*<br><br>*"Doctors have not recommended anything or even told me but I searched for apps and there are too many but I cannot choose which is best. -I did not know about these apps and I did not try to find them." [Focus group1-female-male]*
Expected useful features of smartphone apps	*"Everything will be documented. It helps them [patients] to adhere in taking medications and so on [….] apps have reminders that help to increase patients' participation in managing their health, to make decisions for themselves, and pushing them to do what they always miss, for example, it will remind them to self-monitor." [D8-female]*<br><br>*"Apps allowed recording, BP data where patients usually at hospital visits discuss their BP data verbally with their doctors … they will be involved in their own health decision-making." [D4-female]*<br><br>*FG1P3: Based on what you say, [if these kinds of apps help us to record our data], this app helps us to understand our blood pressure status and to share it with doctors" [Focus group1-male]*<br><br>*"I think it becomes clear or clearer when presented on a graph, but is it measured enough to plot on a graph?"* [D1-male]

	"If apps have information, it will enhance knowledge. We educate them and they absolutely will forget what I said. So, if the information is written in this app, they can read anytime." [D9- female]
Factors affecting uptake of the app	*"I think it depends on the age; much more difficult for older generations to use them" [D2-male]*<br><br>*"If there is good app, I will use it because it encourages us to use especially if it has good interface/menu." [Focus group1- female]*<br>*"I read daily news on my phone, I always zoom out the font to be very clear to read, so I suggest apps should have this feature" [Focus group4-female]*<br><br>*"…apps should be in Arabic because there are not many Arabic apps available" [D12-male]*<br>*"When I searched in apps stores there is not app supported hypertension in Arabic language" [Focus group2- male]*
Concerns about using health apps for self-management	*"I am concerned that the new apps are not yet tested and may be removed [from the app store]. " [D7-male]*<br><br>*"I tried to download apps, however, information seems to be inaccurate (not 100% correct), with practice guidelines etc. …. The credibility of app content is very important. I can ensure credibility for patients if it is based on scientific research or checked by other doctors" [D10-female]*
**Adequacy of app content**	

Feedback & tracking progress	*"I liked the second one [Cora app]. It is easy … and represents important data [BP readings] in the chart and has good quality graph" [D8-female]*<br>*"It's difficult for them [patients] to understand and browse the diagram/chart in the third app [Braun] It's more elaborate .. there is a lot of detail on the chart. So, I don't think it's as practical as the last one [Cora]. " [Focus group3-male]*<br><br>*"When a patient records their reading [in Cora], and sees their average… (e.g., normal), which it is difficult to calculate with manual BP reading, Definitely, they will be enthusiastic to be in this healthy state". [D9-female]*<br><br>*"It [ESH] includes BMI, as it is positively influenced BP control" [D1-male]*<br><br>*"I only like classifying blood pressure readings into stages that may help. [Qardio]" [Focus group1-female]*<br><br>*"I see more detailed feedback [Cora app] compared with other apps [that only use color codes] that may encourage patients to involve themselves more in the management of hypertension" [D12-male].*
Reminders	*"I think I like this app [ESH], but I feel it just focuses on monitoring and reminders for medication - there is no reminder for self-monitoring or for walking that we need with our busy lives. [Focus group 2-female].*<br><br>*"This app [Cora] helps patients to set different activities or challenges to remind them. It is really helpful." [D6-female]*<br><br>*"I like the reminder feature [in Braun], but it does not allow you to enter the title of the reminder so you can know if this is a reminder for medication or for self-monitoring of blood pressure." [Focus group1-male]*

	"It is good to remind patients about his medication time [in ESH], because some patients take different two or three medications. The times patients find it difficult that they take all of them at once, each 24 throughout the day, and this app can remind them about the doses they are needed." [D6-female]
Information provided	*"I totally agree I do not think this app [Qardio] is useful as you notice this app does not offer educational information" [Focus group2-female]*<br><br>*"But It [Qardio] does not offer any information for patients as other apps" [D6-female]*<br><br>*"I also think this app [Cora] has valuable information; it offers sufficient information about BP complications, how to measure BP - our people really need that." [D10-female]*<br><br>*"The patient would be more confident and secure [using the Hyten app] because they know information about the medication they will take, and there is a lot of literature there, even about the illness - there is an explanation of the types of hypertension in all those things." [D6-female]*<br><br>*"Too much detail will make the patients uncomfortable … even if they start to use it [Hyten], they will not regularly use it or adhere to medication" [D5-female]*

User data collected	*"A feature detailing factors that affect hypertension [Qardio] should be included, which concerns the welfare and health care of the patients. This would be a more holistic approach" [D7- male].*<br><br>*"For me I think the details [of entering] does not matter, but the method of data input [in Hyten] is not organized, which make difficult to follow." [Focus group1-male]*<br><br>*"When I enter the diet, I have to enter one to five. So, if I eat rice, vegetables, fruit with dessert, will I enter 1 or 5? It is not clear.*<br>*I agree with you --- It is a very important point that needs to be considered. [Focus geoup1-female---male]*<br><br>*"The app should support somethings like exercise, and add to it matters that fit our patients' needs such as swimming, walking and so on, which is determined in same the previous app [Cora]. It also should add features that meet our patients' needs like Saudi food including rice, Arabic bread and dates and other fatty food or carbohydrate, as most hypertensive patients can benefit of these things." [D8-female]*<br><br>*"Entering data of food is somehow vague, it allows patient to assess and chooses their eating between healthy and unhealthy, but patients mostly do not know. It may misevaluate themselves" [D5-female]*<br><br>*"This app [ESH] is easy to use; the method of inputting data may lead to some mistakes, for example if he enters a wrong name for a medication reminder and takes it [the medication] what will happen" [D12-male]*

Social Support & Content Credibility	*"I think it [Qardio] helps you to keep in touch with your friends or family –--I think keeping in contacting with families or friends is not helpful because many social media allow you. We can just take screen shot of the readings and share it through social media. [Focus group1-male---female]*<br><br>*"The information it presents is very organized. Moreover, I can see that this app is based upon the guidelines of medical organizations, which enhances its credibility. Credibility could also be checked by other doctors or medical companies providing a review of this app; or by additional reviews by other users whom I can trust. [D8-female]*
**App Usability**	
How easy to use	*"I feel this app [ESH] is excellent and icons/menus pane are well organized. At the beginning, we find a menu for monitoring blood pressure and medication separately. This presents app sections clearly." [Focus Group2-male]*<br><br>*"Accessing reminders [of Braun app] is not easy at all, I have to go through many windows so as to set the reminder and it is not clear if it is specific for medication or self-montring." [D12, male, 57]*
Training	*"I think it is likely that patients who used to use apps don't need training and patients who don't use apps will sneed it, this depends on the experience of the patient." [D1-male]*<br><br>The most common opinion about suitable training is that it should be sufficient to teach users how to use the app.<br><br>*"Training should be explained by physicians/educator/researcher for patients how to use it, then it can be used by patients" [D6-female]*

	"Any method of training, for example, instructions on a paper or simple sessions helped a lot in how to use the app" [Focus group4-male]
**Potential benefits and drawbacks of app use**	
Support patients' self-managment	*"Benefits of using this app [Cora] are really good or great; this is a monitoring application that you can customize the application to help patients monitoring themselves, so for example if the patient forgets to take medication or go to walk, the reminder will help them. For this, it is a very good potential tool in managing hypertension." [D7-male]*<br><br>*"This app [Qardio] will help me as doctor not patient." [D9-female]*<br><br>*"The app [Braun] should support somethings like exercise… as I think people may get bored when use this app and reduce their engagement in managing hypertension." [D8-female]*
Expected risks of inappropriate content	*"The detailed medical information [of Cora] is not needed. They[patients] may be get stressed" [D6-female]*<br><br>*"However, if you notice the method of setting the reminder for medication [of Hyten] is not easy to access, despite of providing the list of medicines, this may lead to confuse and reduce their motivation" [Focus Group2-male]*

	"Too much medical details [of Hyten], there is no need for all of these medical details. On the contrary, it may affect negatively.<br>Researcher: How?<br>You know that doctors give the patient the information that he needs to know, but in some cases, you can give them too much information, it may cause an obstacle in taking their drug. What I mean is sometimes we need to reduce the information given to patients in order to avoid confusing them; they just need to know what we want them to know." [D2-male, 36]
**Overall app assessment**	
Factors affecting uptake and usage	*"[T]he difficulty also lies in old-aged people, even if they can read and write." [D3-female]*<br><br>*"If someone who does not own a smart phone were to use it, they would find it more difficult."[D2-male]*<br><br>*"I have concern when we using any of these app may cannot do all tasks efficiently, we need a lot of help and practice before use it." [Focus group4-female]*<br><br>*"It is important to promote about this app[Cora] in media or advertisements by Ministry of Health to support using this app by patients, because doctors alone are not enough to support." [D9-female, 28]*<br><br>*"If apps are for free, it will be great --- Yeah, some people also did not use MasterCard to pay, so they will not download this app." [Focus group3-male---male]*<br><br>*"I don't think they will accept [personal data being uploaded to the server]. If you mention this, [patients] will refuse out of principle" [D2-male]*

	"No any concerns regarding patients' confidentiality, because only the patient will use it and all the entered information isn't sensitive and confidential." [D3-female]<br><br>*"The app language is still a limitation" [Focus Group3-female]*
