# Supplementary material for: Identification of the Most Suitable App to Support the Self-Management of Hypertension: Systematic Selection Approach and Qualitative Study
Source: JMIR Mhealth Uhealth. 2021 Nov 17;9(11):e29207. doi: 10.2196/29207 (PMC8663499; doi:10.2196/29207)
Supplement: Multimedia Appendix 4 [file mhealth_v9i11e29207_app4.docx]

|  | **Cora** | **ESH** | **Hyten** | **Qardio** | **Braun** |
| --- | --- | --- | --- | --- | --- |
| **Theme: Adequacy of app content** | | | | | |
| **Feedback** and **tracking progress** | Participants felt this app contained good quality graphs, color-coded feedback supplemented with text, and offered BP average readings. However, one doctor preferred that the app should offer more direct feedback. | Patients felt that the quality of graphs in this app were good, and doctors liked the BMI feature. However, doctors felt this app lacks feedback in general. | Patients found the graphs unclear in this app. Although feedback was color coded, doctors felt that it should be more directed and linked with age. | Patients found the graphs difficult to understand in this app. Doctors liked the BMI function and the color-coded feedback, but felt it should be linked with age and more directed. | Patients felt the color-coded feedback should be more accurate and directive. Although doctors liked the app presenting lifestyle choice data on charts, they felt that the graphs were not easy to understand and should include more relevant data. |
| **Information provided** | Generally, doctors were satisfied with the level of detail of the information provided. Both doctors and patients liked information about correctly monitoring BP, complications, and general information about the disease. Doctors liked the information provided about medication and side-effects. | Generally, doctors felt that the level of information was unsatisfactory. They believed information about complications and medication was lacking. Patients liked the information about the disease in general. | Doctors liked the variety of information provided. However, doctors and patients felt the information was too detailed and complex. | Participants considerer is not useful due to absence of education. | Doctors suggested that they liked the information relating to lifestyle. However, they felt the app lacked medication data. |
| **User data collected** | Most doctors felt Cora’s health was helped by entering data and tracking BP and medication, which can be entered easily. However, 2 doctors asked to consider weight. Patients and some doctors felt it is unique for allowing patients to enter different tasks, stress levels and sources. | Doctors felt ESH is good for entering BP and weight, but the method of manually typing is a limitation. Some doctors and patients said it lacked relevant lifestyle data. | Doctors and some patients liked the app’s support for medication doses, BP and feelings. Doctors disagreed over the symptom tracking feature: one favored it and another found it unhelpful.  Some patients also found the app’s support for symptoms unhelpful. Some patients found the data inputting to be disorganized. Furthermore, the app does not consider the weight. | Both patients and doctors like the method of entering weight. Doctors said it lacked support for inputting and tracking other lifestyle factors such as diet and, medication other conditions. | Doctors liked the app’s inclusion of BP and a lifestyle tracking feature, but they found this inaccurate and too general. They also noted that the app also does not support weight or medication names. Patients also agreed that despite considering lifestyle, the method of entering data is not helpful. |
| **Reminder** | Generally, most doctors and patients were satisfied with Cora as it provided reminders. Some doctors said that it provided the best reminders among all the apps. | Patients and doctors were satisfied with the reminders for medication, with a small number of doctors (n=2) liking its inclusion of different reminders for different medication and dosage. A few doctors and patients would have liked it to include reminders for other tasks | Doctors were satisfied with the reminders for medication and few liked the inclusion of reminders for different medication. A few doctors would have liked it to include more reminders. Patients also like the reminder allowing the input of medication name and dose, but some think the reminder setting is not easy. | Doctors felt that Qardio is good to have a reminder for BP, but they also would like reminders for other tasks including medication. Some patients felt the reminder is too generic. | Doctors felt that Braun is good to have reminder, but this is only very simple. They would like reminders for other tasks. Some patients found that the reminder feature of Braun too generic. |
| **Social support** |  |  |  | Patients have mixed opinions about the importance of social support feature; some find useful and other not. Two doctors felt this feature encourages patients to use this app. |  |
| **Content Credibility** | Doctors felt that the credibility of educational information, and the references of the readings, could be checked.  They expressed that the credibility of the information could be assessed and that it could be based upon medical guidelines. They also suggested that the app could be reviewed by additional doctors and medical companies. Finally, they asked whether the purpose of the app was to help people or gain profit? | | | | |
| **Theme: App Usability** | | | | | |
| **How easy to use** | Most doctors (n=12) and patients agreed that Cora is easy to use and practical because its interface is easy to navigate and data entry is easy as well as having friendly colours. | Most participants in interviews (n=10) and focus groups agreed that ESH care app is easy to use. Patients felt it was friendly and had simple colors. Very few patients also felt that navigation would be easier if features were organized horizontally. | Many doctors (N=8) and some patients in focus groups found this app easy to use because it simply requires clicking and selecting and having user-friendly colors. However, some agreed that its navigation is somewhat complex. | Opinions were mixed about app usability. Some doctors (n=6) agreed it is generally easy to use due requiring fewer actions/tasks, while most participants in all focus groups felt it is not easy to use due to its unclear layout affecting navigate | Most participants agreed that Braun is not user-friendly (n=8), and most participants in focus groups believed it was difficult to use because of its annoying color, poor layout, and the difficulty of entering data. |
| **Training** | 3 doctors and some patients said patients would not need any training to use Cora, except older people. Other doctors and patients believed it would need around one training session, or another resource, e.g. a leaflet with instructions, to teach them how to use it. | 4 doctors said that patients did not need any training to use it if it will be in Arabic. Other doctors said it would need around one training session. Patients had mixed opinions: some said it need training for anywhere between one session and up to three weeks | Two doctors said it did not need any training. Others said it may need training based on user medical knowledge and app background. Patients agreed that training would be needed, either face-to-face or via video tutorials. | Two doctors said the app would need training. Others said it would need a leaflet, video tutorial or manual guidance.  Most patients said it would need training, but there was disagreement about whether this would need to be simple or intensive. | Doctors and patients said that Braun would need training but they disagreed about whether this would need to be intensive or simple, and about how many sessions. |
| **Theme: Overall app assessment** | | | | | |
| ***Doctors’ willingness to recommend apps*** | Most doctors (N=10) are willing to recommend this app and describe it as ‘complete’ or ‘comprehensive’ due to its detailed functions e.g,. BP average and multiple reminders. | More than half of doctors (n=7) are willing to recommend it due to features such as its consideration of BMI and its high-quality information. | Most Doctors (n=9) are willing to recommend it and describe it as complete, due to its detailed information. The rest think the level of information is too detailed for patients | One doctor is willing to recommend this app. A  small number of doctors were willing to recommend it on account of specific features, but not on its overall functionality. More than half of doctors (n=8) believed that Qardio is less recommendable because it focuses mostly on monitoring BP, and lacks other useful feature, like education. | Two doctors are willing to recommend this app,  with a small number of doctors being willing to recommend it on account of specific features, but not on its overall functionality. More than half of doctors (n=8) believed that Braun is less recommendable because its features, e.g. reminders, lack detail, and because of the difficulty of entering data. |
| ***Patients’ willingness to use and recommend app to other*** | Most patients in all focus groups stated they would use Cora and would recommend it to friends, as it is easy to use in terms of entering data, and they liked the quality of feedback (e.g. graphs), and features such as suitable information, tracking and reminders. | Most patients in focus groups stated they would use ESH care, and would recommend it to friends, due it being easy to use, and providing good information and tracking. | Two groups were positive about the app, due to its support for inputting medication names and feelings, and the information provided. The other two groups were more negative, due to e.g. excessive level of data requested, and low-quality charts. | Focus groups’ responses to Qardio were mostly negative, due to lack of information provided, poor quality charts, and its limited tracking. However, one patient approved of Qardio’s support for communication with family and friends. | Focus group responses to Braun were mostly negative, due to issues such as its limited reminder feature and the complexity of its layout and data input. |
| ***Doctors’ Estimated uptakes*** | Doctors expected that the majority of patients (around 80% to 90%) would use it to help manage their disease. | Doctors expected that around 60% to 70% of patients would use it to help manage their disease. | Doctor expected that a good percentage of patients (around 50%) would use it. | Doctors expressed that few patients would use it. | Some doctors felt it would be used by around 30% to 40% of patients. However, one doctor felt that no patient will use it. |
| ***General recommendations*** | One doctors asked to add the word ‘high and low’ on the chart showing BP. | Doctors suggested the addition of more detailed information and reminders and considerations of other lifestyle factors. They also suggested inputting data by selecting rather than manually typing.  Patients asked for more reminders and consideration other factors that affect BP. | Some doctors suggested the addition of more daily tips or reminders about exercise, and for feedback to be more sensitive to age. Patients asked to improve the charts to be clearer and the set reminder to be easier and for the app to provide audio reminder. | Doctors suggested the addition of more reminders, an education feature and for feedback to be more customized. They also suggested the app consider other factors affecting BP, as well as medication. Patients suggested considering other life style factor and adding messaging with a doctor in emergency cases. | Doctors and patients asked to improve the method of entering lifestyle data to make this more accurate. They also suggested the inclusion of more reminders for exercise and healthy diet, and for simplified graphs. |
|  | A few doctors recommended the addition of other features for all five apps such as adding hospital appointments, doctor access and other medical conditions. | | | | |
| **Factor affecting uptake and usage** | Two doctors think the app is more suitable for younger users. Two doctors stressed the need for practice for older patients who do not have IT background before. | Two doctor felt that ESH is more suitable for older people especially who are accustomed to smartphone technology. Some Patients felt that this app is more suitable for people who do not adhere in taking medication. | Some doctors expressed that this app is more suitable for younger or people who have previous experience with IT. Some patients felt | Some doctors expressed that this app is more suitable for people who have controlled BP or are looking only for self-monitoring BP. | Some doctors expressed that technical difficulties may be a main barrier for users. |
|  | For all five apps, some patients expressed that the cost of apps and the payment method required may be a difficulty for some patients. Some patients felt using | | | | |
|  | For all five apps, most patients and doctors considered language to be a main barrier to patient uptake, i.e. the apps being unavailable in Arabic. | | | | |
|  | For all five apps, there was mixed opinion amongst doctors and patients regarding the importance of privacy. One doctor felt privacy was important, and another doctor believed privacy is important in the case of high-profile individuals. One doctor stressed the importance of a passcode to protect app data. Most patients did not express concern with the confidentiality of the apps. | | | | |
|  | Two doctors stressed the importance of official endorsement as a factor affecting patient uptake. They felt patients would be more likely to use apps that were supported by e.g. the MOH or mentioned in public health campaigns. | | | | |
| **Theme: Potential benefits and drawbacks of app use** | | | | | |
| **Potential benefits and drawbacks of app use** | Most doctors and patients felt Cora helped people to: control BP, save time and share information with their doctor during appointments. It also helped to engage patients, increase compliance to different tasks and awareness, due to it having a BP average and a tracker that tells you how many tasks are completed.  One doctor felt this detailed information may cause stress for patients. | Most doctors felt that ESH may enhance hospital follow-up and the sharing of data. They also stated that it can increase patient involvement and awareness by monitoring BP and weight, as well as educational information.  However, less features may lead the patient to become bored. | Doctors felt that detailed education and medication reminders, in addition to monitoring may increase patient enthusiasm and compliance to take medication. It may also increase their awareness and ability to share data with doctors and control BP.  However, patients and doctors felt too much detailed information and the way of setting reminders may confuse some patients and distract them. | Some doctors felt that Qardio may help to increase awareness of why BP is low or high through monitoring, adherence and Feedback. Others also felt this app is more helpful for doctors at follow up visits, rather than patients. However, too few features may lead patients to get bored and decrease their use. | Some doctors felt that Braun may increase health awareness and encourage people to control BP, due to education and monitoring. Some Patients also felt that considering factors may increase enthusiasm. However, some doctors felt that inaccurate inputs and the low-quality of charts may reduce app use, or lead to boredom and confusion in patients. |
